# Vasculitis syndromes: large vessel vasculitides and ANCA-associated vasculitides from a neuroradiologist’s perspective

**DOI:** 10.1007/s11604-025-01896-1

**Published:** 2025-10-30

**Authors:** Koichi Takano, Kosuke Hida, Tomonobu Tani, Kengo Yoshimitsu

**Affiliations:** 1 Department of Radiology, Fukuoka University Chikushi Hospital, Chikushino, Japan; 2 Department of Radiology, Faculty of Medicine, Fukuoka University, Fukuoka, Japan

**Keywords:** Takayasu arteritis, Giant cell arteritis, ANCA-associated vasculitis, Granulomatosis with polyangiitis, Microscopic polyangiitis, Eosinophilic granulomatosis with polyangiitis, Hypertrophic pachymeningitis

## Abstract

The 2022 version of the American College of Rheumatology (ACR)-European Alliance of Associations for Rheumatology (EULAR) classification criteria for large vessel vasculitis and antineutrophil cytoplasmic antibody (ANCA)-associated vasculitides (AAVs) was substantially updated from the previous 1990 ACR criteria, with the role of diagnostic imaging significantly increased in accordance with the development of imaging modalities and expanding knowledge of the imaging findings. This review illustrates the basic characteristics of vasculitis syndromes in adults involving large vessels such as Takayasu arteritis, giant cell arteritis, and AAVs, with a brief introduction to the updated 2022 ACR/EULAR classification criteria, along with key clinical and imaging clues from a neuroradiologist’s perspective.

## Introduction

The 2022 publication of the American College of Rheumatology (ACR)-European Alliance of Associations for Rheumatology (EULAR) classification criteria for large vessel vasculitis [1, 2] and antineutrophil cytoplasmic antibody (ANCA)-associated vasculitides (AAVs) [3–5] was a substantial renewal from the previous 1990 ACR criteria [6–9] which had been widely utilized for decades but were recently criticized for their insufficient performance in differentiating disease entities [10, 11]. With the updated criteria, the role of diagnostic imaging has significantly increased in accordance with advances in imaging modalities and improved understanding of the findings.

This review illustrates the basic characteristics of these vasculitis syndromes in adults involving large vessels such as Takayasu arteritis (TAK), giant cell arteritis (GCA), and ANCA-associated vasculitides including granulomatosis polyangiitis (GPA), microscopic polyangiitis (MPA) and eosinophilic GPA (EGPA). A brief introduction to the new 2022 ACR/EULAR classification criteria is provided, along with key clinical features and recent advances in imaging from a neuroradiologist’s perspective. Table 1 summarizes the principal intracranial and head and neck imaging findings of each disease.

**Table 1:** principal intracranial and head and neck imaging findings in large vessel vasculitis and ANCA-associated vasculitis

	Large Vessel Vasculitis		ANCA-associated Vasculitis		
	TAK	GCA	GPA	MPA	EGPA
Intracranial	Infarction	Arteritis -ICA, VA Infarction	Hypertrophic pachymeningitis Small vessel diseases/cerebrovascular events		
Extracranial-neck vessels	Arteritis - CCA, VA - US macaroni sign	Arteritis -STA, OA, VA -US halo sign -DWI scrolling artery sign			
Orbital		Arteritis -OpA (AION)	Granuloma		
Sinonasal			Rhinosinusitis Granuloma Bone destruction Septal defect		Rhinosinusitis -CT hyperdense
Large vessels	Arteritis -Ao, SCA, AxA, PA -Stenosis/occlusion -Subclavian steal	Arteritis -Ao, SCA, AxA			

*TAK* Takayasu arteritis, *GCA* Giant cell arteritis, *GPA* Granulomatosis with polyangiitis, *MPA* Microscopic polyangiitis, *EGPA* Eosinophilic granulomatosis with polyangiitis, *ICA* Internal carotid artery, *VA* vertebral artery, *CCA* Common carotid artery, *SCA* Subclavian artery, *STA* Superficial temporal artery, *OA* Occipital artery, *US* Ultrasound, *OpA* Ophthalmic artery, *AION* Anterior ischemic optic neuropathy, *Ao* Aorta, *AxA* Axillary artery, *PA* Pulmonary artery.

## Updated 2022 ACR/EULAR criteria

The new 2022 ACR-EULAR classification criteria for the two large-vessel vasculitides (LVVs) and three AAVs are shown in Tables 2, 3, 4, 5, and 6. Details of each criterion are discussed in the following sections. It should be noted that each criterion has two entry requirements: (a) These classification criteria should be applied when a diagnosis of vasculitis has been made, and (b) alternate diagnoses mimicking vasculitis should be excluded prior to applying the criteria. It should also be noted that, in contrast to the previous 1990 criteria, each item has a differently weighted score, and some items negatively impact the total score in the new 2022 criteria.

**Table 2:** 2022 ACR/EULAR classification criteria for Takayasu arteritis

Absolute requirements	
Age ≤60 years at time of diagnosis	
Evidence of vasculitis on imaging	
Additional Clinical Criteria	
Female Sex	+1
Angina or ischemic cardiac pain	+2
Arm or neck claudication	+2
Reduced pulse in upper extremity	+2
Vascular bruit	+2
Carotid artery abnormality*	+2
SBP difference in arms ≥20 mmHg	+1
Additional Imaging Criteria	
Number of affected arterial territories (select one)	
One arterial territory	+1
Two arterial territories	+2
Three or more arterial territories	+3
Systemic involvement of paired arteries	+1
Abdominal aorta with renal or mesenteric involvement	+2

A score of ≥5 is needed for the classification of Takayasu arteritis

^*^Reduction or absence of pulse of the carotid artery or tenderness of the carotid artery

Adapted from [1]

**Table 3:** 2022 ACR/EULAR classification criteria for giant cell arteritis

Absolute requirements	
Age ≥50 years at time of diagnosis	
Additional clinical criteria	
Morning stiffness in shoulders/neck	+2
Sudden visual loss	+3
Jaw or tongue claudication	+2
New temporal headache	+ 2
Scalp tenderness	+2
Abnormal examination of the temporal artery*	+2
Laboratory, Imaging, and Biopsy Criteria	
Maximum ESR ≥50 mm/hour or CRP ≥10 mg/liter	+3
Positive temporal artery biopsy or halo sign on ultrasound	+5
Bilateral axillary artery involvement**	+2
FDG-PET activity throughout aorta	+2

A score of ≥6 is needed for the classification of giant cell arteritis

^*^Examination of the temporal artery showing absent or diminished pulse, tenderness, or hard ‘cord-like’ appearance

^**^Defined as luminal damage (stenosis, occlusion, or aneurysm) on angiography (computed tomography, magnetic resonance, or catheter-based) or ultrasound, halo sign on ultrasound or FDG uptake on PET

Adapted from [2]

**Table 4:** 2022 ACR/EULAR classification criteria for granulomatosis with polyangiitis

Clinical criteria	
Nasal involvement: bloody discharge, ulcers, crusting, congestion, blockage, or septal defect/perforation	+3
Cartilaginous involvement (inflammation of ear or nose cartilage, hoarse voice or stridor, endobronchial involvement, or saddle nose deformity	+2
Conductive or sensorineural hearing loss	+1
Laboratory, Imaging, and Biopsy Criteria	
Positive test for cANCA or PR3-ANCA*	+5
Pulmonary nodules, mass, or cavitation on imaging	+2
Granuloma, extravascular granulomatous inflammation, or giant cells on biopsy	+2
Inflammation, consolidation of the nasal/paranasal sinuses, or mastoiditis on imaging	+1
Pauci-immune glomerulonephritis on biopsy	+1
Positive test for pANCA or MPO-ANCA**	−1
Blood eosinophil count ≥1 × 10^9^/liter	−4

A score of ≥ 5 is needed for the classification of Granulomatosis with Polyangiitis

^*^Cytoplasmic antineutrophil cytoplasmic antibodies or antiproteinase 3 antibodies

^**^Perinuclear antineutrophil cytoplasmic antibodies or antimyeloperoxidase antibodies

Adapted from [3]

**Table 5:** 2022 ACR/EULAR classification criteria for microscopic polyangiitis

Clinical criteria	
Nasal involvement: bloody discharge, ulcers, crusting, congestion, blockage, or septal defect/perforation	−3
Laboratory, imaging, and Biopsy Criteria	
Positive test for pANCA or MPO-ANCA*	+6
Fibrosis or interstitial lung disease on chest imaging	+3
Pauci-immune glomerulonephritis on biopsy	+3
Positive test for cANCA or PR3-ANCA**	−3
Blood eosinophil count ≥1 × 10^9^/liter	−1

A score of ≥5 is needed for the classification of microscopic polyangiitis

^*^Perinuclear antineutrophil cytoplasmic antibodies or antimyeloperoxidase antibodies

^**^Cytoplasmic antineutrophil cytoplasmic antibodies or antiproteinase 3 antibodies

Adapted from [4]

**Table 6:** 2022 ACR/EULAR classification criteria for eosinophilic granulomatosis with polyangiitis

Clinical criteria	
Obstructive airway disease	+3
Nasal polyps	+3
Mononeuritis multiplex	+1
Laboratory and Biopsy criteria	
Blood eosinophil count ≥1 × 10^9^/liter	+5
Extravascular eosinophilic-predominant inflammation on biopsy	+2
Positive test for cANCA or PR3-ANCA*	−3
Hematuria	−1

^*^Cytoplasmic antineutrophil cytoplasmic antibodies or antiproteinase 3 antibodies

Adapted from [5]

A score of ≥6 is needed for the classification of Eosinophilic Granulomatosis with Polyangiitis

## Large vessel vasculitis

### Takayasu arteritis (TAK)

TAK is a major form of LVV primarily affecting the aorta, its branches and pulmonary arteries [4, 12, 13]. TAK is more common in Asia and predominantly affects young women, and the reported median onset age is 35 years [13].

Symptoms vary and may reflect, either systemic or vascular inflammation and organ ischemia including brain. Head and neck complaints rank second to systemic manifestations and include dizziness/vertigo, neck pain (carotidynia) and headache [13, 14].

In the updated 2022 ACR/EULAR classification criteria (Table 2), the absolute requirements are: (a) age at the diagnosis ≤60 years, and (b) imaging evidence of vasculitis. In addition to classic findings, (a) female sex, (b) angina, (c) arm or neck claudication, and (d) carotid artery abnormality are newly included after the ACR 1990 criteria [1].

**Fig. 1:**
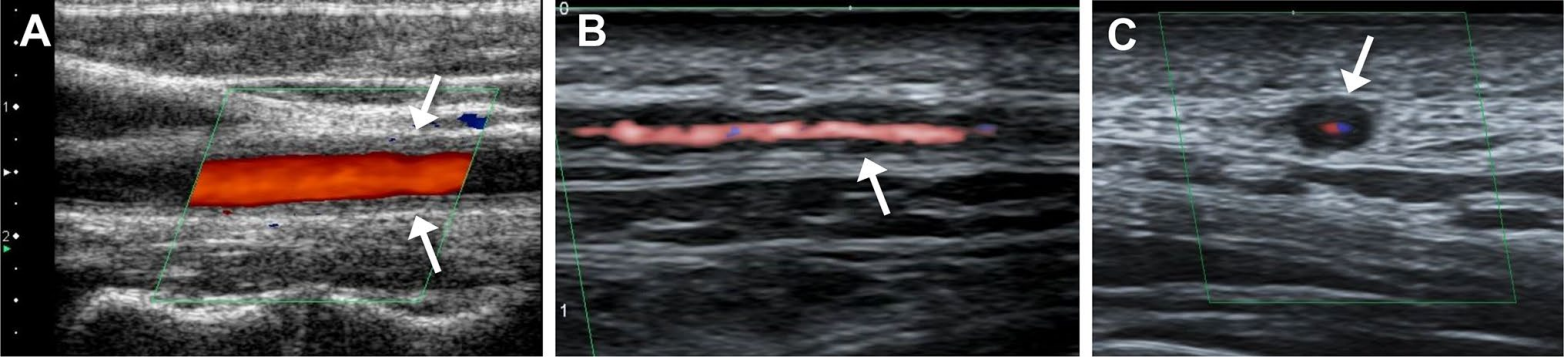
Ultrasound in Takayasu arteritis (TAK) (**A**) and giant cell arteritis (GCA) (**B**) and (**C**). (**A**) Long axis image of the common carotid artery in a 36-year-old woman with TAK shows echogenic wall-thickening (macaroni sign, arrows). (**B**) Long axis and (**C**) short axis images of the temporal artery in a 57-year-old man with GCA show circumferential hypoechoic wall-thickening (halo sign, arrows)

On ultrasonography (US), homogenous circumferential thickening of the common carotid artery walls has been described as the “macaroni sign” (Fig. 1A) [15, 16]. Similar findings may be observed in other large arteries such as the subclavian and axillary arteries. The arterial wall appears midechoic, in contrast to the hypoechoic “halo sign” in GCA (Fig. 1B, C).

**Fig. 2:**
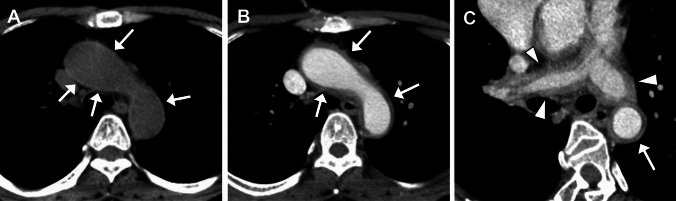
Double-ring sign in Takayasu arteritis. A 49-year-old woman with back pain. (**A**) Pre-contrast CT shows hyperattenuation along the aortic wall (arrows). (**B**) Contrast-enhanced CT shows hyperdense outer (arrows) and hypodense inner layers of the aortic wall. (**C**) An oblique short-axis image of aortic arch clearly shows a concentric ‘double-ring’ appearance (arrow). Note diffuse wall thickening of the pulmonary arteries (arrowheads)

Currently, contrast-enhanced (CE) CT (CE-CT) is widely used to evaluate luminal and mural changes in the affected arteries. A typical manifestation in early-stage TAK is concentric mural thickening [17–19]. The walls of affected arteries often show hyperdense on unenhanced CT (Fig. 2A) [17, 18]. CE-CT, particularly late phase rather than early arterial phase, often reveals a double ring enhancement pattern in the walls of affected arteries (Fig. 2B, C). The well-enhanced outer ring is believed to represent active inflammation of the media and adventitia, whereas the poorly enhanced inner ring reflects gelatinous swelling of the intima [17, 18].

Pulmonary arteries are involved in 20–70% of patients with TAK [18, 20, 21]. CE-CT reveals circumferential wall-thickening with enhancement, with or without luminal narrowing (Fig. 2C). Usually, double ring features and calcifications are not observed [17].

**Fig. 3:**
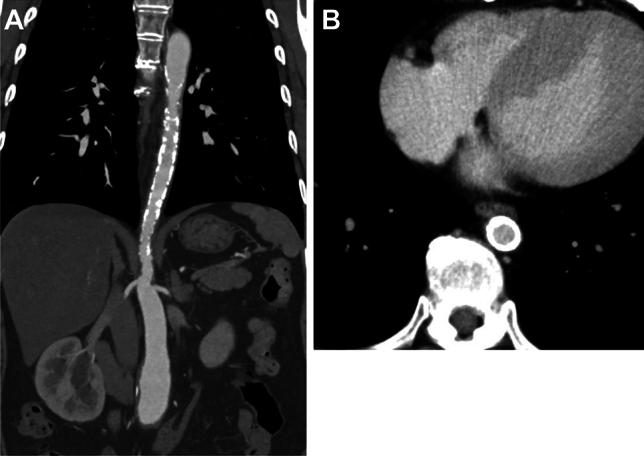
Contrast-enhanced CT in chronic-phase Takayasu arteritis in a 34-year-old woman. (**A**) Coronal image shows long-segment, rat tail or radish tail-like narrowing with wall calcification of the descending aorta, and mild dilatation of the lower abdominal aorta. (**B**) Axial CT shows concentric, full-thickness wall calcification of the narrowed descending aorta

In the late phase of TAK, arterial stenoocclusive (and/or less commonly, aneurysmal) changes can be observed. A smooth distal tapering, often called as “rat tail” or “radish tail” aorta, is typical (Fig. 3A) [19]. Wall calcification, which is typically circumferential, is commonly observed (Fig. 3B). Calcification is mostly transmural, in contrast to subintimal calcification in atherosclerosis [18].

**Fig. 4:**
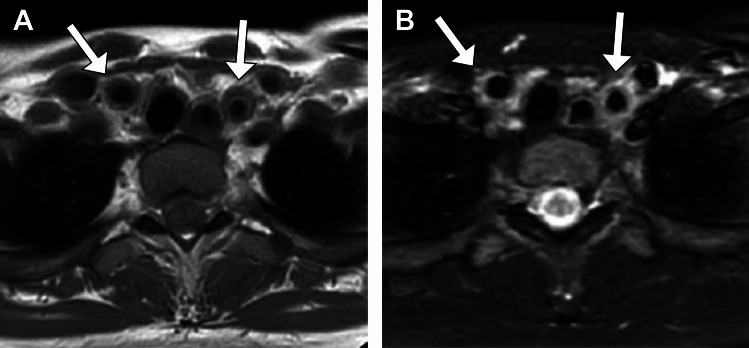
MRI in an 18-year-old woman with Takayasu arteritis. (**A**) T1WI and (**B**) STIR show wall thickening of the left and right common carotid arteries with increased signal of the wall and surroundings on STIR (**B**) (arrows)

MRI is also useful for early diagnosis because of its ability to evaluate wall thickness, T2-hyperintensity and contrast enhancement of not only the walls but also the surrounding tissue (Fig. 4) [22, 23]. In addition, low *b*-value diffusion-weighted imaging (DWI) has been reported to be a possible alternative to CE-T1-weighted image (T1WI) for detecting inflammation [24]. In the 2023 EULAR recommendations, MRI is positioned as the first imaging test for patients with suspected TAK [25]. Although some authors have suggested that MRI findings reflect the disease activity, considerable overlap remains between the active and inactive phases, and the role of MRI in evaluating disease activity remains limited [23].

**Fig. 5:**
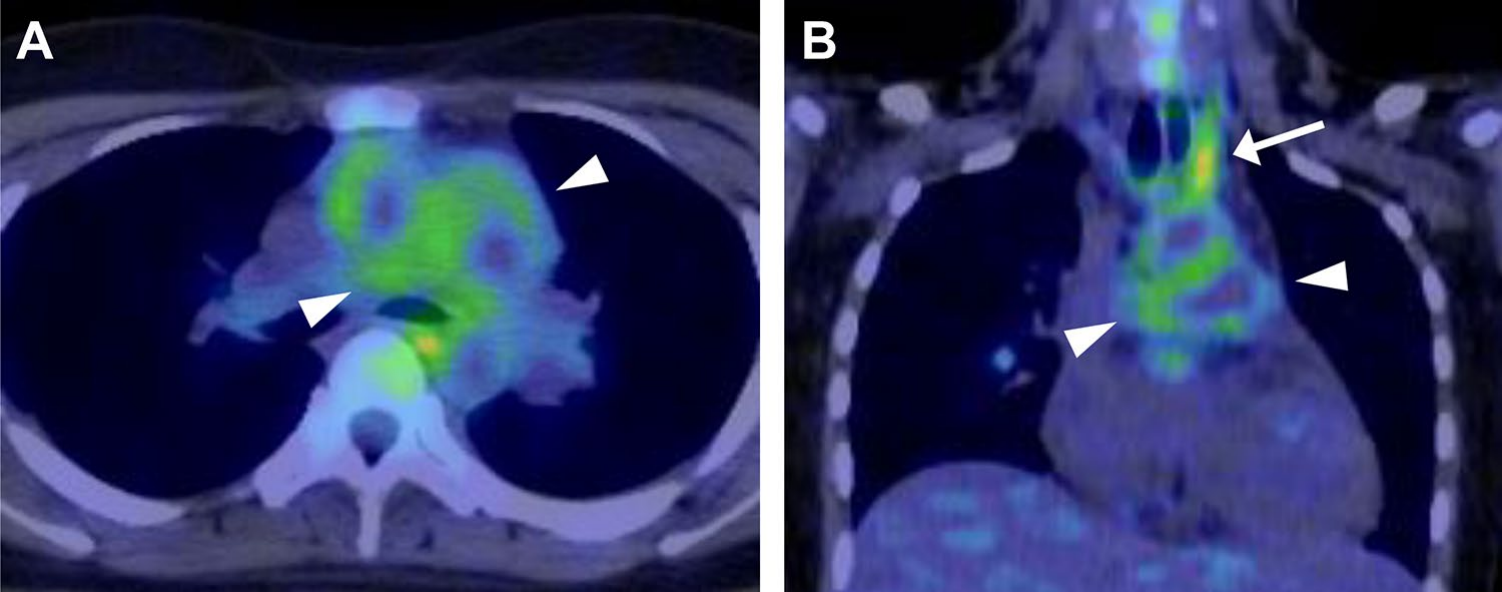
Fused FDG-PET/CT in Takayasu arteritis. A 18-year-old woman (same patient as in Fig. 4). (**A**) Axial and (**B**) Coronal images show FDG uptake in the aorta and its major branches, particularly the left common carotid artery (arrow), and pulmonary arteries (arrowheads)

The use of ^18^F-fluorodeoxyglucose (FDG)-positron emission tomography (PET) for LVV was approved under the Japanese national health insurance system in 2018 [26]. Many studies have indicated that FDG-PET is useful for diagnosing LVVs [27–29]. The predominant uptake in positive scans is linear and conforming to the arterial wall, although the uptake is not always visually conspicuous (Fig. 5) [27, 28]. FDG-PET detects TAK earlier and more specifically than MRI [29, 30]. However, the role of FDG-PET in the evaluation of disease activity remains unclear. Although association between FDG uptake and disease activity markers has been observed, poor correlation has also been reported [31]. The routine use of FDG-PET for follow-up evaluation of LVV is not recommended in the 2023 EULAR recommendations [24].

**Fig. 6:**
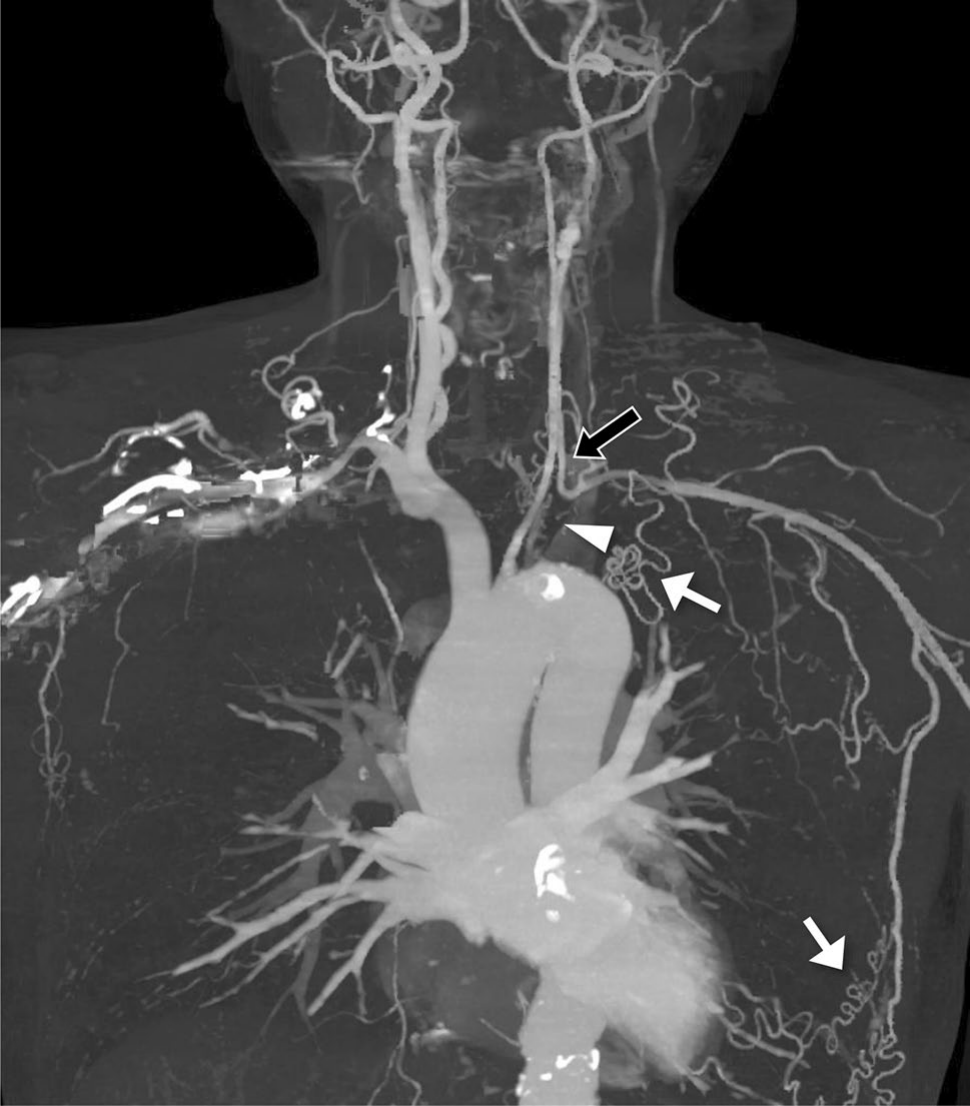
Subclavian steal in chronic-phase Takayasu arteritis in a 41-year-old woman. MIP of CT-angiography shows occlusion of the proximal portion of the left subclavian artery (arrowhead) with distal filling via the left vertebral (black arrow) and the intercostal arteries (white arrows)

In TAK, incidence of brain ischemia is reported as 7–11% of the patients [14, 32, 33]. Imaging may reveal occlusive changes in the common carotid, subclavian, vertebral, and intracranial arteries. In one series of 79 patients with TAK, almost a quarter had a subclavian steal (Fig. 6) [32]. Reports of TAK with other cerebrovascular diseases include intracerebral [34] and subarachnoid hemorrhage [35], posterior reversible encephalopathy syndrome (PRES) [36], reversible cerebral vasoconstriction syndrome [37], and moyamoya disease [38].

**Fig. 7:**
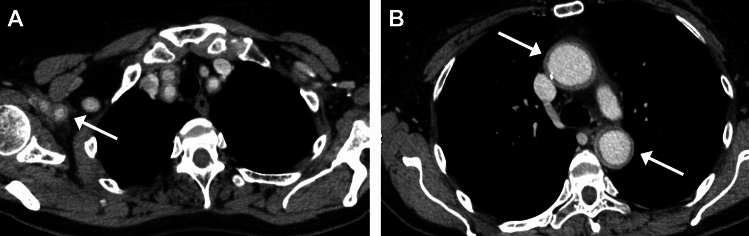
Large vessel involvement in giant cell arteritis. A 67-year-old woman with fever. Contrast enhanced CT shows wall-thickening and enhancement of the large arteries including the right axillary artery (arrow in **A**). The thickened aortic wall shows ‘double-ring’—like appearance (arrows in **B**)

Findings indicating or mimicking aortitis including ‘double ring’ appearance can be observed in other entities such as GCA (Fig. 7), AAVs, Behçet disease, relapsing polychondritis, sarcoidosis, IgG4-related disease (IgG4-RD), and infectious aortitis [39, 40].

### Giant Cell Arteritis (GCA)

GCA, formerly known as temporal arteritis, is the most common form of systemic vasculitis in patients aged ≥50 years, with an incidence peak in persons aged between 71 and 80 years [2, 41, 42]. GCA is defined as granulomatous arteritis that affects large- and medium-sized arteries, with a predisposition to affect the extracranial branches of the carotid arteries [12]. Its prevalence in Japan is low compared with that in other countries [42].

Common presenting features include new headache, jaw claudication, scalp tenderness, and visual disturbances [2, 7]. It may associate polymyalgia rheumatica although the incidence of this association is low, approximately 30% in Japan [42].

In the updated 2022 ACR/EULAR classification criteria (Table 3), the age at the time of diagnosis ≥50 years is the absolute requirement. In addition to the classic manifestations, (a) morning stiffness in shoulders/neck, (b) sudden visual loss, (c) jaw or tongue claudication, and (d) abnormal examination of the temporal arteries were newly included after the ACR 1990 criteria. In the laboratory, imaging and biopsy criteria, (a) halo sign of temporal artery on US, (b) bilateral axillary involvement on imaging and (c) FDG-PET activity throughout aorta were newly added [2].

The halo sign, which is investigated by color Doppler US and is defined as a homogeneous, hypoechoic and concentric thickening of the arterial wall (Fig. 1B, C) and not compressible, has a sensitivity and specificity of 68 and 81%, respectively [43]. The halo sign may also be observed in other involved vessels, such as the occipital and axillary arteries [44].

**Fig. 8:**
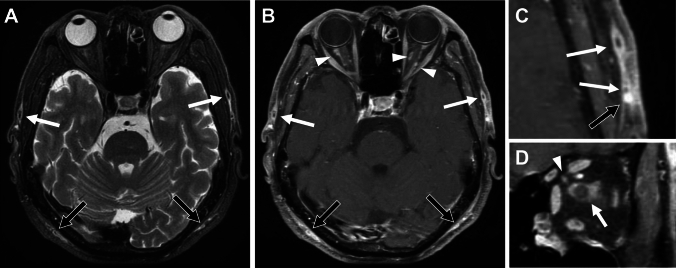
Giant cell arteritis. A 75-year-old man with acute left visual disturbance. (**A**) Fat-suppressed (FS) T2WI shows hyperintensity and (**B**) FS contrast-enhanced (CE) T1WI shows enhancement of the walls of the temporal (white arrows) and occipital arteries (black arrows) as well as surrounding tissue. Note enhancement surrounding the bilateral optic nerves (arrowheads in B). (**C**) Magnified image of (**B**). Note strong vascular enhancement posterior to the artery represents superficial temporal vein (black arrow in C). (**D**) Coronal FS CE-T1WI of the left orbit. Optic nerve and surrounding enhancement (arrow), and enhancement of the thickened ophthalmic artery (arrowhead)

MRI has been widely used to assess inflammation of the vessel walls in the temporal (and occipital) arteries. High-resolution, gadolinium-enhanced fat-suppressed (FS) T1WI (usually spin-echo type, either 2D or 3D) is mostly used to detect thickening with abnormal enhancement of the arterial wall and surrounding structures (Fig. 8) [45–49]. FS T2WI may also show increased signal (Fig. 8) [50]. In general, evaluation at higher magnet strength is more useful, and 3D vessel-wall imaging sequences perform better than 2D sequences [48, 49]. MRA may or may not reveal luminal stenosis of affected arteries.

**Fig. 9:**
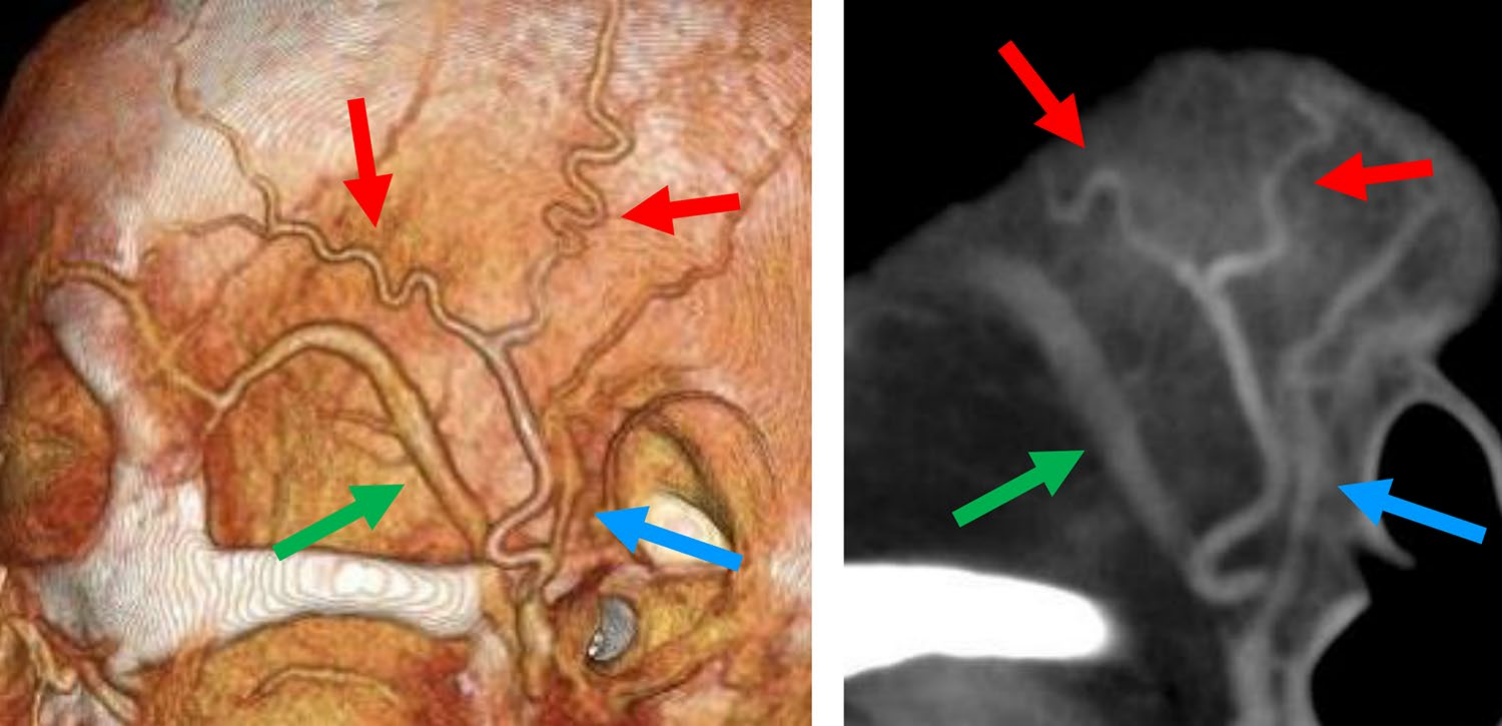
Anatomy of the temporal artery and veins. CT-angiography (**A**) and oblique sagittal reformat of unenhanced CT (**B**). Note the tortuosity and wide branching angle of the temporal artery (red arrows) which can be recognized on sagittal image (**C**) and distinguished from the middle temporal (green arrow) and superficial temporal (blue arrow) veins

**Fig. 10:**
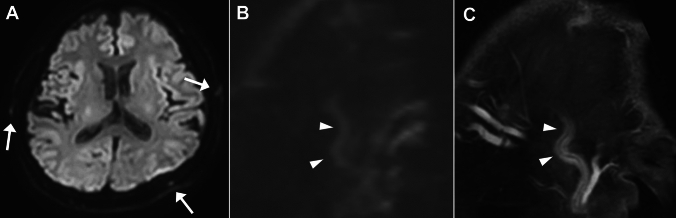
Diffusion-weighted image (DWI) in giant cell arteritis. A 57-year-old man with headache. (**A**) DWI shows hyperintensity of the bilateral temporal and left occipital arteries (arrows). (**B**) Oblique sagittal reconstruction of DWI shows hyperintensity of the temporal artery as seen in wall enhancement on (**C**) fat-suppressed contrast enhanced T1WI (arrowheads in B and C)

Notably, the enhancement and artifact of the veins often mimics arterial enhancement [44]. Understanding the morphological characteristics of the temporal arteries, including tortuosity and branching patterns, may help distinguishing them from the veins. (Oblique) sagittal images are often helpful for evaluating arterial morphology (Figs. 9 and 10) [48].

There is limited literature on cranial CT angiography in GCA. Blurred vessel margins and perivascular enhancement have been reported [51].

DWI findings of the extracranial arteries have been reported as ‘DWI-scrolling-artery sign’ which is defined as extracranial signals that appear as blood vessels when scrolling through DWI images (Fig. 10) with a sensitivity of 73.6% and a specificity of 94.2%. Although its sensitivity is lower than that of CE-vessel wall imaging, this technique is useful because DWI is available in routine non-contrast protocol and can be used immediately in daily practice [52].

**Fig. 11:**
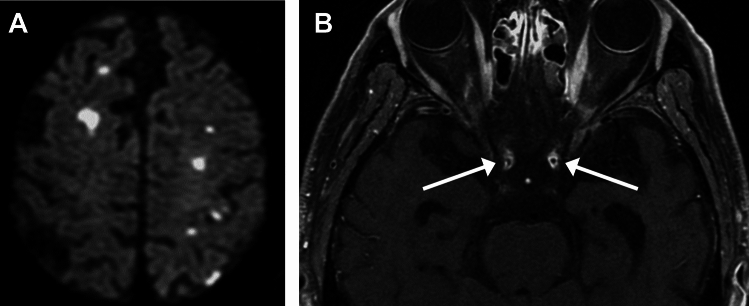
Internal carotid artery involvement and cerebral infarctions in giant cell arteritis. An 80-year-old man with GCA with impaired consciousness. (**A**) DWI shows multiple acute-stage cerebral infarctions bilaterally. (**B**) Fat-suppressed, contrast-enhanced black blood 3D-T1WI shows wall enhancement and luminal narrowing of the bilateral internal carotid arteries (arrows)

In GCA, the intracranial vessels may be affected in approximately 10–50%, with the intradural ICA and vertebral arteries most commonly involved, showing circumferential wall thickening and enhancement (Fig. 11) [44, 47, 49]. Cerebrovascular events have been reported in 2–12% of patients with GCA [44, 47]. Cerebral infarctions tend to be multiple, bilateral, often occur in watershed areas (Fig. 11), and may be refractory [53].

It should be noted that intracranial arterial wall enhancement can be observed in apparently normal and asymptomatic, particularly older individuals. Enhancement is predominant in the vertebral arteries and can be circumferential [54]. Wall enhancement without thickening may be related to normal aging.

Ocular symptoms in GCA are common and most frequently attributed to anterior ischemic optic neuropathy (AION) associated with arteritis of the ophthalmic artery and its branches [44, 55]. On MRI, orbital abnormality is observed in approximately 30% of patients. Enhancement of the optic nerve sheath, surrounding tissue, and intraconal fat is most prevalent. Enhancement of the ophthalmic artery wall can also be observed (Fig. 8) [44, 55].

Optic nerve head enhancement (central bright spot sign) is more frequently observed in GCA-AION than in nonarteritic AION [56]. Similarly, DWI-hyperintensities/diffusion restrictions have also been reported in the optic nerves, particularly at the nerve heads more frequently in arteritic AION than in nonarteritic AION [57].

Additionally, soft tissue abnormalities, such as abnormal enhancement of the temporalis muscle [58] and arcuate STIR-hyperintensities in extracranial superficial soft tissues (Fig. 8) (multifocal arcuate sign) [59] have reported in GCA. Enhancement of the temporalis muscle showed a moderate correlation with jaw claudication [58].

**Fig. 12:**
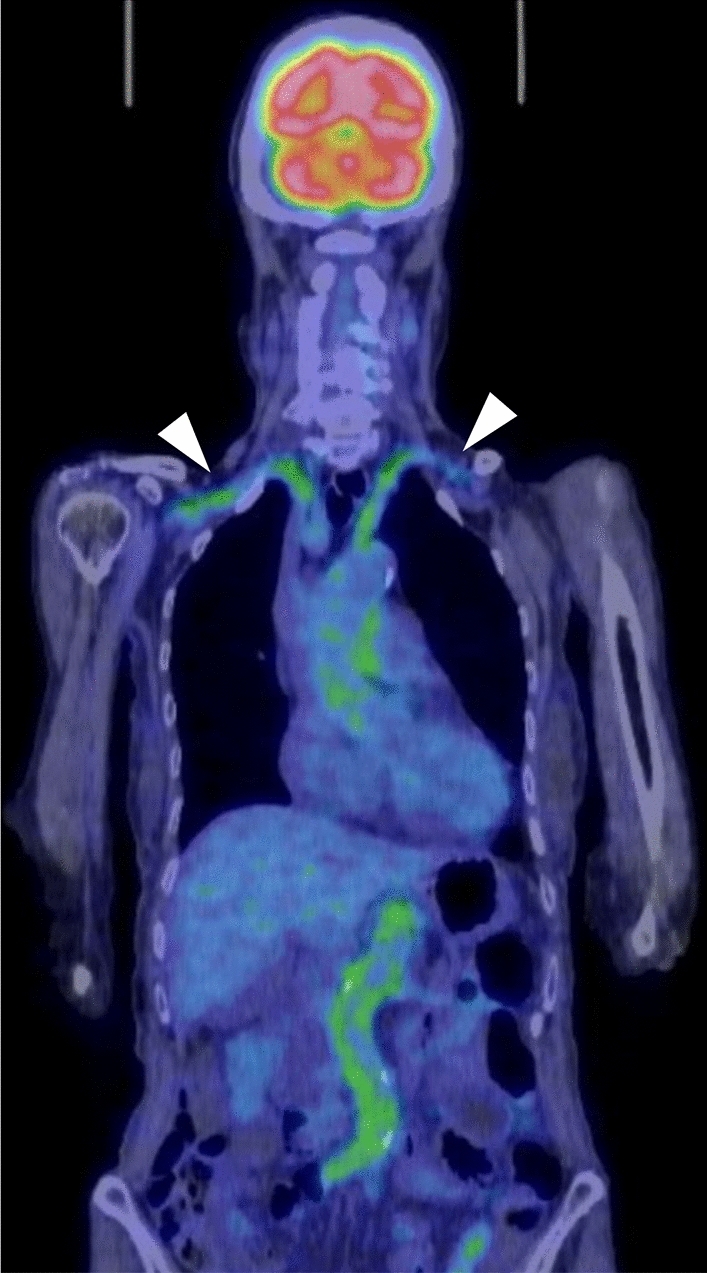
Fused FDG-PET/CT in giant cell arteritis in an 84-year-old woman. Coronal image shows FDG uptake in the aorta and bilateral common subclavian-axillary arteries (arrowheads)

**Fig. 13:**
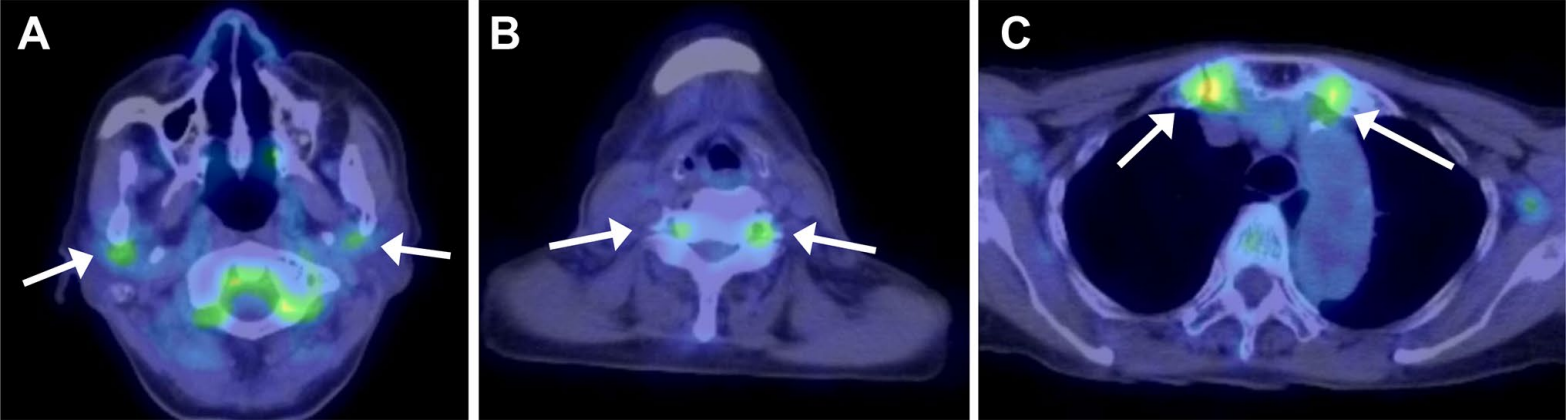
Fused FDG-PET/CT in giant cell arteritis in an 87-year-old woman. Axial images show FDG uptake in the bilateral external carotid arteries (arrows in A) and vertebral arteries (arrows in B). Uptake in the bilateral sternoclavicular joints indicates associated polymyalgia rheumatica (arrows in C)

GCA may involve the aorta and its major branches. The subclavian and axillary arteries, and thoracic aorta are most frequently affected. In a multicenter study, 66% of patients with GCA had at least one large arterial lesion at diagnosis [60]. US, CT (Fig. 7), MRI and FDG-PET [61] are widely used to assess the large vessels in GCA. FDG-PET, as in TAK, is useful for assessing overall extent of vasculitis (Fig. 12), may simultaneously reveal features of polymyalgia rheumatica (Fig. 13), and can exclude the presence of underlying pathologies such as malignancy or infection [44]. The 2023 update to the EULAR guidelines recommends the use of FDG-PET as the preferred technique for evaluating extracranial arteries [24]. In addition, FDG-PET is useful for detecting inflammation of cranial arteries, such as the external carotid branches and vertebral arteries (Fig. 13) [62].

Various entities have been reported to involve the temporal arteries, with atherosclerosis being the most common. Other entities that mimic temporal arteritis include AAVs, amyloidosis, IgG4-RD, infectious diseases, and neoplasms [43, 63].

### ANCA-associated Vasculitis (AAV)

AAV, a systemic small- to medium-vessel vasculitis, is characterized by pathogenic ANCA production and comprises GPA, MPA, and EGPA [12].

GPA is more common in Northern Europe, whereas the occurrence rates of MPA are relatively high in Southern Europe and Japan [64]. In a nationwide cohort study of AAV in Japan, the proportions of GPA, EGPA and MPA (including renal-limited vasculitis) were 21, 9, and 50%, respectively [65].

Neurological involvement is not uncommon in AAV**.** While the peripheral nervous system is commonly affected at a frequency of 20–65%, central nervous system (CNS) involvement is relatively rare, reported in approximately 5–15% [66–68].

Although CNS involvement in AAV varies, some features are shared among the subtypes. Hypertrophic pachymeningitis (HP) is the most frequent CNS presentation. Small vessel diseases (SVD) and PRES may also be observed. Isolated parenchymal mass lesions/granulomas and spinal cord involvement are rarely reported [67, 69].

### Hypertrophic Pachymeningitis (HP) in AAV

HP is a chronic inflammatory disorder characterized by thickened dura mater [66, 70, 72]. In a previous study in Japan, the frequencies of spinal HP, cranial HP, and combined spinal/cranial HP were 9, 85, and 4%, respectively [70].

ANCA-related HP is the most frequent, followed by idiopathic HP. Other causes of HP include IgG4-RD, sarcoidosis, rheumatoid arthritis, Sjögren syndrome, systemic lupus erythematosus, non- Langerhans histiocytosis, and infectious diseases [66, 70–72]. In addition, TAK and GCA have been reported to cause HP [74]. HP affects up to 4.5% of patients with AAV, with GPA being the most common diagnosis [71, 72].

Cranial HP can occur almost anywhere, with no site preference [67]. Among the symptoms, headache is most frequent (>90%), followed by seizures and cranial neuropathy [67, 72].

**Fig. 14:**
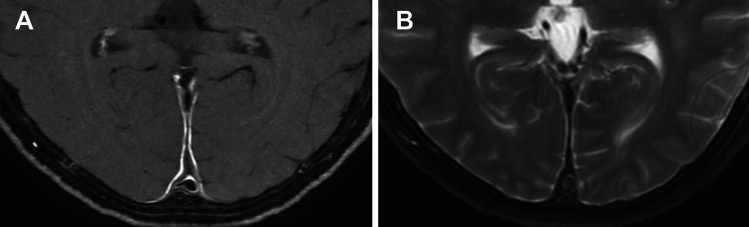
Hypertrophic pachymeningitis (HP) in ANCA-associated vasculitis (AAV) in a 60-year-old woman with MPO-AAV. (**A**) Fat-suppressed (FS) contrast enhanced T1WI (CE-T1WI) shows peripheral pattern enhancement and central hypointensity of the thickened falx. (**B**) FS-T2WI shows central hypointensity

**Fig. 15:**
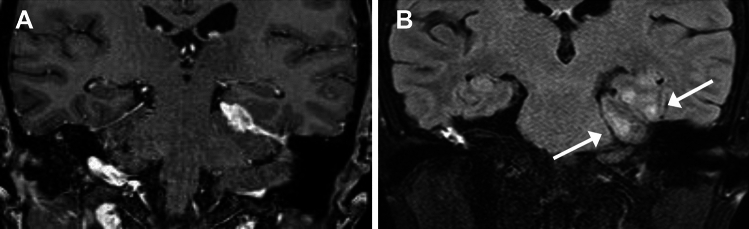
Hypertrophic pachymeningitis (HP) in ANCA-associated vasculitis in a 64-year-old woman with granulomatosis with polyangiitis. (**A**) Coronal FS-CE-T1WI shows HP involving the left side of the tentorium. (**B**) FS-FLAIR shows hyperintensity in adjacent brain parenchyma (arrows)

**Fig. 16:**
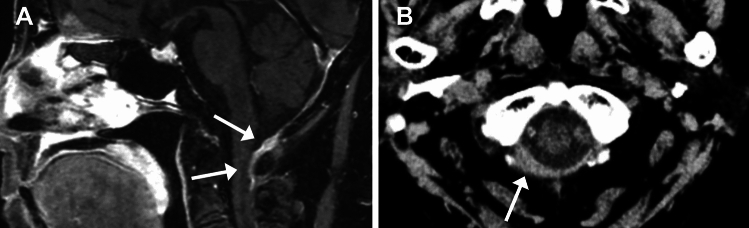
Hypertrophic pachymeningitis (HP) in ANCA-associated vasculitis in a 76-year-old woman with microscopic polyangiitis. (**A**) Sagittal FS-CE-T1WI shows HP at the posterior fossa to upper cervical spine (arrows). (**B**) CT shows hyperdense dural thickening

CE-T1WI reveals enhancement of thickened dura. Enhancement may be homogeneous, or often shows a peripheral dominant pattern with central hypointensity, which is usually hypointense on T2WI and hyperdense on unenhanced CT reflecting dense fibrosis (Figs. 14 and 16). When lesion involving the posterior falx and tentorium is observed on coronal CE-T1WI, it may resemble illuminated Eiffel Tower (“Eiffel-by-night sign”) [67, 71–74]. Associated inflammation may be observed in adjacent leptomeninges or brain parenchyma, particularly in PR3-ANCA-positive patients (Fig. 15) [66, 67].

Differential diagnoses of HP include spontaneous intracranial hypotension (SIH), subdural hematoma, and neoplasms. SIH usually exhibits T2-hyperintensity, broader distribution, and does not show central hypointensity on CE-T1WI, in contrast to HP, although the associated subdural hematoma may complicate findings in SIH. Venous distension is often associated with SIH [75] but is usually not observed in HP.

Recent reports have suggested an association of MPO-ANCA positivity (either MPA or MPO-ANCA-positive GPA) with spinal involvement in HP [72, 76], although its occurrence is infrequent (Fig. 16).

### Small Vessel Diseases (SVD) in AAV

**Fig. 17:**
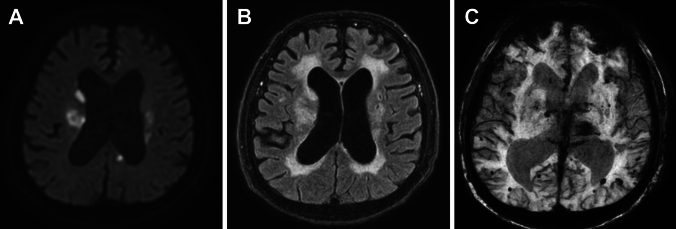
Small vessel diseases in a 71-year-old man with microscopic polyangiitis. (**A**) DWI shows cerebral infarctions. (**B**) FLAIR shows white matter hyperintensity and small old infarctions. (**C**) Susceptibility-weighted image shows microbleeds

SVD/cerebrovascular events may occur to variable degrees in AAVs. Infarctions may present as isolated or multiple lesions predominantly affecting the supratentorial parenchyma (Fig. 17) [67]. Hemorrhagic events occur less often and affect the brain parenchyma or, less frequently, the subarachnoid space [67, 77]. In addition, MRI may reveal nonspecific white matter lesions with T2 hyperintensities, which are difficult to distinguish from atherosclerotic disease (Fig. 17) [67–69].

### Granulomatosis with Polyangiitis (GPA)

GPA, formerly Wegener granulomatosis, is a rare multisystemic disease in the AAV group. The ear, nose, throat, lungs, and kidneys are primarily involved, but almost any organ including the CNS can be affected [3, 12, 69, 78]. Men and women are equally affected, with age at onset 45–65 years and a higher prevalence among white individuals [68].

In the updated 2022 ACR/EULAR classification criteria (Table 4), (a) cartilaginous involvement in ear or nose (such as hoarse voice and saddle nose deformity) and (b) hearing loss, were newly included compared with the 1990 ACR criteria [3, 8]. In the laboratory, imaging and biopsy criteria, along with the classic findings such as pulmonary nodules/mass/cavitation, (a) positive test for cANCA or PR3-ANCA, (b) inflammation of the nasal/paranasal sinuses, or mastoiditis on imaging and (c) pauci-immune glomerulonephritis on biopsy were newly added [3].

Neurological involvement in GPA occurs in 22–54%, with peripheral neuropathy being the most common [69, 79]. CNS involvement is rarely seen, occurring in 3–12% of patients, and the most common form is HP (Fig. 15) [69, 79]. Other patterns of CNS involvement include SVDs and leptomeningeal infiltration from surrounding organs. Primary intraparenchymal granuloma is extremely rare [69, 79].

**Fig. 18:**
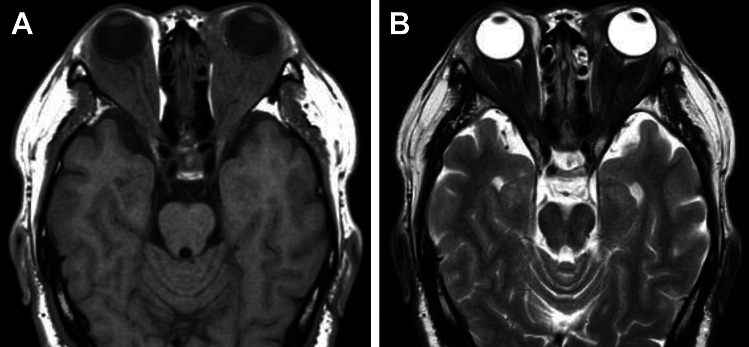
Orbital involvement in a 56-year-old man with granulomatosis with polyangiitis. (**A**) T1WI and (**B**) T2WI show diffuse intraorbital infiltrate with T1- and T2-hypointensity

Ophthalmologic manifestations are common, occurring in 45–58% of patients with GPA [69, 80]. Orbital masses are more frequently unilateral, extraconal or trans spatial in distribution, and often coexist with sinus disease [69, 80]. A diffuse inflammatory infiltrate that molds to the contour of the orbit is commonly observed, and is typically hypointense on T1WI and T2WI (Fig. 18) [69, 80].

Several conditions can mimic orbital disease in GPA, including IgG4-RD, sarcoidosis, and lymphoma. Sarcoidosis and lymphoma often cause concomitant involvement of the lacrimal gland [80].

The nose and paranasal sinuses are commonly involved in GPA, affecting 29–36% of patients [72]. In the early stage, GPA frequently manifests as a nonspecific chronic sinusitis pattern, and imaging may not enable differentiation between mucosal inflammation and granuloma; granuloma typically shows hypointensity on both T1WI and T2WI, and nodular thickening may suggest GPA [69, 80, 81].

**Fig. 19:**
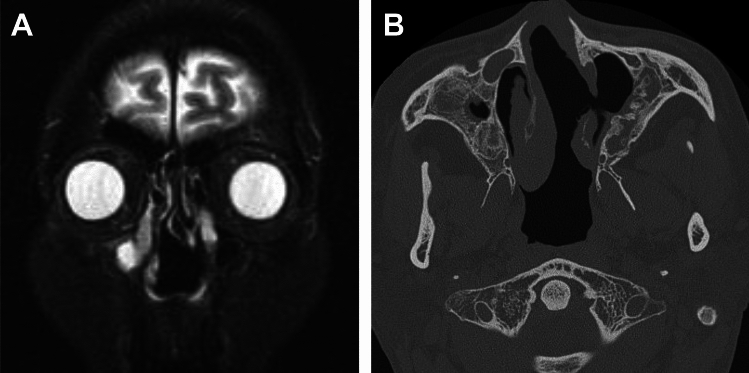
Sinonasal involvement in a 50-year-old man with granulomatosis with polyangiitis. (**A**) Coronal STIR shows septal defect and maxillary sinus hyperintensity. (**B**) CT shows destruction of the nasal septum and left inferior concha, as well as neoosteogenesis of the maxillary sinuses

CT is helpful in evaluating bone alterations. The combination of osseous destruction and neoosteogenesis favors GPA (Fig. 19). Destruction of cartilage and bone typically begins at the septum and turbinates and spreads symmetrically, eventually resulting in a single cavity. The hard palate is characteristically spared [69, 80, 81]. Eventually, saddle-nose deformities, present in 10–25% of patients, result from nasal collapse [69, 80, 81].

The sinonasal features of GPA may mimic cocaine necrosis, sarcoidosis, and lymphoma. Hard palate defects are common with lymphoma but they are distinctly uncharacteristic of GPA [79]. Sarcoidosis can be indistinguishable from GPA; however, nasal septal destruction is not a characteristic feature of sarcoidosis [79].

Otologic manifestations occur in up to 40% of patients with GPA, most frequently associated with serous otitis media. In addition, enhancement of adjacent cranial nerves (most frequently the facial nerve) or the cochlea (labyrinthitis) may be observed on MRI. [69, 79].

Airway involvement is found in up to 55% of patients with GPA, typically occurring as a late complication. The imaging features are nonspecific, revealing inflammatory mucosal thickening, which may lead to circumferential subglottic stenosis in the late stage [69, 79]. The major differential diagnoses include amyloidosis, tracheal neoplasm, sarcoidosis, relapsing polychondritis, and postintubation tracheal stenosis [79].

### Microscopic Polyangiitis (MPA)

MPA, which was once recognized as a particular type (microscopic form) of polyarteritis nodosa, was not included in the 1990 ACR criteria. In the 2012 revised International Chapel Hill Consensus Conference nomenclature of vasculitides [12], MPA is separated from PAN and included in the AAV group. MPA is a necrotizing vasculitis predominantly involving small- to medium-sized arteries linked to MPO- ANCA and is accompanied by pauci-immune glomerulonephritis and interstitial lung disease (ILD) [4, 12, 64, 77].

MPA predominantly affects middle aged and older patients, with ages at onset of 55–70 years. Men and women are equally involved. Main clinical symptoms include renal manifestations, mononeuritis multiplex, and lung involvement [77, 81]. ILD is common and typically precedes diagnosis [64].

In the updated 2022 ACR/EULAR classification criteria (Table 4), (a) positive test for pANCA or MPO-ANCA, (b) fibrosis or ILD on imaging, and (c) pauci-immune glomerulonephritis on biopsy were assigned 6, 3, and 3 points, respectively. In contrast, (d) nasal involvement, (e) positive test for cANCA or PR3-ANCA and (f) elevated blood eosinophil count were assigned negative points [4].

As with other subtypes of AAV, MPA cause CNS impairments much less frequently than peripheral neuropathy [68, 82]. In a series of 85 patients with MPA, mononeuritis multiplex was observed in 57.6%, whereas only 11% showed CNS involvement [85].

CNS involvement in MPA other than HP includes parenchymal mass lesions, PRES, cerebral infarction, intracerebral and subarachnoid hemorrhages [67, 77], white matter hyperintensities and microbleeds, suggesting an increased SVD burden (Fig. 17) [82, 84, 85].

### Eosinophilic Granulomatosis with Polyangiitis (EGPA)

EGPA (formerly Churg-Strauss syndrome) is a rare form of AAV characterized by eosinophil-rich granulomatous inflammation and small to medium-sized vessel vasculitis associated with bronchial asthma, sinonasal inflammation, peripheral neuropathy, and eosinophilia [5, 86, 87]. Adult-onset asthma is a key manifestation, occurring in more than 90% of patients [86, 87]. The median age at onset is 49–59 years, with no extreme gender bias. Eosinophil counts are mostly elevated and ANCA (usually MPO-ANCA) is positive in 30–47% of patients [86].

In the updated 2022 ACR/EULAR classification criteria (Table 6), (a) asthma (b) paranasal sinus abnormalities and (c) neuropathy, mono or poly in the old criteria [9] are now superseded by (a) obstructive airway disease, (b) nasal polyps and (c) mononeuritis multiplex. Positive test for cANCA or PR3-ANCA had a negative impact [5].

EGPA causes CNS impairments much less frequently than peripheral neuropathy [68, 86]. Peripheral nerve manifestation is frequent (51–98%) and commonly presents a mononeuritis multiplex pattern [86, 88].

CNS involvement in EGPA has been reported in 5–29% of cases. The main CNS manifestations include HP, ischemic cerebrovascular disease, intracerebral and/or subarachnoid hemorrhage [88], and PRES [89]. Imaging abnormalities in the intracranial vessels are usually not evident, although abnormal DSA findings have been infrequently reported [90].

In EGPA, chronic rhinosinusitis/nasal polyp is common initial symptom which is present in 48–86% of cases, and typically precedes diagnosis. Patients with EGPA have nasal polyps more frequently than those with GPA [24, 26, 91–94].

**Fig. 20:**
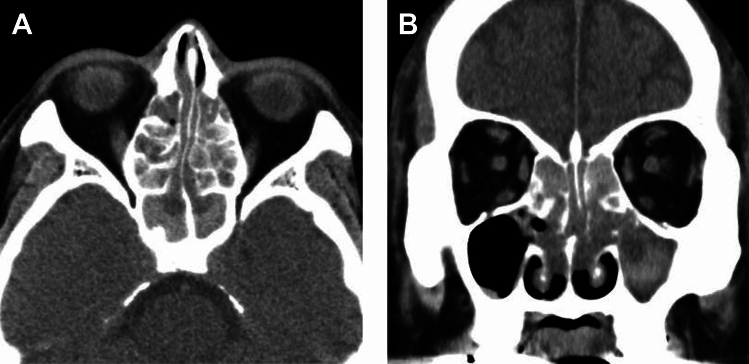
Sinonasal involvement in a 62-year-old man with eosinophilic granulomatosis with polyangiitis. (**A**) Axial and (**B**) coronal CT show diffuse sinus opacity with hyperdense areas, and nasal polyps

Ryoo et al. reported that more patients with EGPA showed high-density (>60 HU) sinus opacification (Fig. 20) than those with GPA. High CT attenuation, similar to that in allergic fungal sinusitis and eosinophilic chronic rhinosinusitis, is presumably attributable to allergic mucin with high protein concentrations [94]. In contrast, GPA showed more bone destruction, including nasal septa (45%), bone sclerotic changes (Fig. 19), and involvement of surrounding organs, whereas 9% of patients with EGPA also showed nasal septal destruction [94].

## Conclusion

The role of diagnostic imaging increased significantly in the updated 2022 ACR/EULAR classification criteria for LVVs and AAVs. However, CNS and head and neck imaging diagnoses of these syndromes are often challenging as the findings are diverse, occasionally nonspecific, often subtle, and can be readily overlooked in routine clinical practice. Radiologists should therefore consider the possibility of vasculitis syndromes and be aware of the above-mentioned intracranial and extracranial findings, such as the scalp, to improve the detection and diagnosis of LVVs and AAVs.
